# A comparison of metal/metal and ceramic/metal taper‐trunnion modular connections in explanted total hip replacements

**DOI:** 10.1002/jbm.b.34897

**Published:** 2021-06-23

**Authors:** Eileen S. Cadel, L.D. Timmie Topoleski, Oleg Vesnovsky, Charles R. Anderson, Robert H. Hopper, Charles A. Engh, Matthew A. Di Prima

**Affiliations:** ^1^ US Food and Drug Administration Silver Spring Maryland USA; ^2^ University of Maryland, Baltimore County Baltimore Maryland USA; ^3^ Anderson Materials Evaluation, Inc. Columbia Maryland USA; ^4^ Anderson Orthopaedic Research Institute Alexandria Virginia USA

**Keywords:** ceramic‐on‐metal, metal‐on‐metal, taper‐trunnion connection, total hip arthroplasty explants

## Abstract

Corrosion and wear are commonly found at the taper‐trunnion connection of modular total hip arthroplasty (THA) explanted devices. While metal/metal (M/M) modular taper‐trunnion connections exhibit more wear/corrosion than ceramic/metal (C/M) modular taper‐trunnion connections, damage is present in both, regardless of material. This study used a combination of assessment techniques including clinical data, visual scoring assessment, optical imaging, profilometry, and x‐ray photoelectron microscopy (XPS), to investigate wear mechanisms and damage features at the modular taper‐trunnion connection of 10 M/M and 8 C/M explanted THAs. No correlation was found between any demographic variable and corrosion wear and assessment scores. All assessment techniques demonstrated that the stem trunnions had more damage than head tapers for both explant groups and agreed that C/M explants had less corrosion and wear compared to M/M explants. However, visual assessment scores differed between assessment techniques when evaluating the tapers and trunnions within the two groups. Profilometry showed an increase (*p* <.05) in surface roughness for stem trunnions compared to head tapers for both explant groups. X‐ray photoelectron spectroscopy performed on deposits from two M/M explants found chromium and molybdenum carbides beneath the surface while chromium sulfate and aged bone mineral were found on the surface suggesting that the debris is a result of corrosion rather than wear. These results indicate that taper‐trunnion damage is more prevalent for M/M explants, but C/M explants are still susceptible to damage. More comprehensive analysis of damage is necessary to better understand the origins of taper‐trunnion damage.

## 1 INTRODUCTION

Biological issues with wear performance and response to metal wear debris led the Food and Drug Administration to reclassify total hip arthroplasty (THA) implants with metal‐on‐metal bearings as Class III medical devices^1^ based on the recommendation from an Orthopaedic and Rehabilitation Devices Panel. While there are currently no FDA‐approved metal‐on‐metal THAs marketed for use in the United States, the use of metal heads on metal stems that creates a metal/metal (M/M) taper‐trunnion modular connection still allows for wear/corrosion at the modular interface which has led to clinical reports of adverse local tissue reactions (ALTRs) in THAs without metal‐on‐metal bearings.^2^, ^3^, ^4^, ^5^ Ceramic heads, made either from alumina or zirconia‐toughened aluminum, on metal stems create a ceramic/metal (C/M) taper‐trunnion modular connection and have been shown to reduce the amount of wear and corrosion at the modular taper connection^6^, ^7^ resulting in their use as an alternative to metal heads.^8^, ^9^ The potential for wear/corrosion between the ceramic head and the metal stem remains due to the difference in hardness between metals and ceramics, and thus further characterization is still needed.

Damage of modular head tapers has been assessed using a number of metrics including visual assessment,^10^, ^11^, ^12^ volume loss measurements,^6^, ^12^ mass loss measurements,^13^, ^14^ as well as electrochemical assessment of the contacting surfaces.^13^, ^14^ Visual assessments are generally qualitative and are based on the subjective judgment of multiple observers who rate the visual or photographic surfaces of the modular connections and provide a score based on the relative quantity and intensity of the observed wear/corrosion on the surfaces.^10^, ^11^, ^12^ However, scoring methods with different scales and scoring criteria can make it difficult to compare results across different studies. Volume loss measurements are more quantitative than the visual assessment and use coordinate measurement machines to measure changes in the taper/trunnion volume compared to the ideal or as‐manufactured volume.^6^, ^12^ Mass loss is a quantitative assessment where components are weighed before and after testing to measure the amount of material removal from the contacting surface.^13^, ^14^ Measuring the electrochemical potential while the modular surfaces are dynamically loaded allows for a direct measurement of corrosion and re‐passivation of the modular surfaces.^13^, ^14^ Of the four techniques, visual assessment and volume loss are most suited for assessing explanted modular head tapers since lack of preimplantation mass data and tissue fixation can prevent accurate mass measurements of the explanted devices and it is not possible to perform electrochemical measurements of the modular head tapers once explanted. While many of these previous studies have shown a correlation between the different metrics, the need for multiple metrics underscores the complexity of assessing the performance of modular connections.

Previous studies have provided significant insight into the performance of modular taper‐trunnion connections in THA and there have been a number of studies characterizing M/M taper‐trunnion performance. A study investigating titanium stems coupled with titanium adapter sleeves showed that explants with severe head taper corrosion had greater bearing wear and elevated chromium and cobalt serum ion levels.^15^ A study on explanted cobalt chromium heads on metal stems showed no difference in fretting and corrosion as a function of head taper size.^16^ Fretting corrosion comparisons between cobalt chromium heads and titanium or cobalt chromium stems showed significantly more material loss in cobalt chromium stems.^17^ For ceramic heads, previous research has shown that mechanically assisted crevice corrosion occurs to a lesser extent, and the wear rate is an order of magnitude lower: 0.01 mm^3^/10^6^ cycles for ceramic head tapers with metal stem trunnions versus 0.1 mm^3^/10^6^ cycles for M/M taper‐trunnion devices.^18^ This has been attributed to better mechanical interlocking of contact surfaces, which reduced micromotion, as well as the ceramic heads having greater hardness than their metal counterparts. However, due to the paucity of studies involving ceramic head tapers with metal stem trunnions, these findings cannot necessarily be generalized to more than one brand of hip implant, nor can significant connections be drawn between ceramic head taper corrosion and patient‐related factors, such as implantation time.^7^, ^9^

The goal of this study was twofold: (a) to investigate the differences in the wear mechanisms and corrosion behavior of the modular connection for M/M and C/M head tapers and stem trunnions using a combination of qualitative and quantitative techniques including clinical data, visual scoring assessments, optical imaging, profilometry, and x‐ray photoelectron spectroscopy (XPS) of explanted THAs, and (b) to combine information from different qualitative and quantitative assessment techniques to provide a more comprehensive comparison between M/M and C/M taper‐trunnion modular connections. Pairing de‐identified clinical data with these techniques allowed for the comparison of differences between clinical and material factors at the modular taper‐trunnion connection. This study was based on detailed assessment of 18 explants, 10 with M/M modular taper‐trunnion connections and 8 with C/M modular taper‐trunnion connections.

## 2 MATERIALS AND METHODS

### 2.1 Explants

A total of 10 M/M and 8 C/M retrieved THA devices were evaluated for this study. All devices were obtained during revision surgeries at the Anderson Orthopedic Clinic (Alexandria, VA). Of the 18 explants, 10 were DePuy Pinnacle systems (5 Prodigy stems, 3 AML stems, 1 Corail stem, and 1 Tri‐Lock stem), 2 were DePuy Duraloc with AML stems, 2 were Biomet Monobloc Cup with Taperloc stems, 2 were Stryker Trident with Accolade stems, 1 was Wright Conserve Plus with a Profemur Plasma stem, and 1 was Zimmer Continuum Trabecular Metal with an M/L stem. The stem and head material for all 10 M/M explants and the stem for the C/M explants were cobalt chromium molybdenum alloy (CoCrMo). All M/M explants had metal‐on‐metal bearings while all C/M explants had ceramic‐on‐polyethylene bearings. The CoCrMo heads ranged from 36 to 42 mm in diameter, and the ceramic heads ranged from 28 to 36 mm in diameter.

All devices were cleaned by soaking in enzymatic solution to remove most organic material and disinfected in 10% formalin solution and then stored at the Anderson Orthopedic Research Institute (Alexandria, VA). The experimental procedures were approved by the institutional review boards at both the Anderson Orthopedic Clinic and the U.S. Food and Drug Administration.

Infection, loosening, fracture, dislocation, ALTR, and pain were listed as the causes of revision for these 18 patients. Demographic and explant data are presented in Table 1. Of the 10 M/M explants, 6 were from male and 4 were from female patients, with ages ranging from 36 to 63 years at the time of surgery. Patient BMI ranged from 24 to 48 and implant time in vivo ranged from 0.08 to 6.5 years. Of the 8 C/M explants, 4 were from male and 4 were from female patients with ages ranging from 22 to 78 at the time of surgery. Patient BMI ranged from 23 to 46 and implant time in vivo ranged from 0.08 to 12.2 years. The explants were selected for this study because they had both the femoral stem (trunnion) and head (taper) components, and they represented the full range of wear and corrosion at the taper interface on visual inspection (little to no wear/corrosion to obvious evidence of wear/corrosion).

**TABLE 1:** Clinical and explant information

	Metal/metal modular connection	Ceramic/metal modular connection
Number of explants	10	8
Gender (M:F)	6:4	4:4
Age at implantation (years)		
Average ± *SD*	53 ± 8.8	54 ± 20
Range	(36–63)	(22–78)
BMI		
Average ± *SD*	31 ± 7.8	34 ± 10
Range	(24–48)	(23–46)
Time in vivo (years)		
Average ± *SD*	2.5 ± 2.1	2.8 ± 4.3
Range	(0.08–6.5)	(0.02–12.2)
Reason for revision:		
Infection	3	3
Loosening	3	2
Bone fracture	2	1
Dislocation	0	2
ALTR	1	0
Pain	1	0
Cup abduction angle (°)		
Average ± *SD*	44.4 ± 5.8	46.0 ± 9.0
Range	(39–56)	(34–58)
Cup anteversion angle (°)		
Average ± *SD*	19.1 ± 12.1	25.2 ± 8.8
Range	(1–35)	(15–38)

### 2.2 Corrosion and wear mechanisms assessment

#### 2.2.1 Visual scoring methods

**FIGURE 1:**
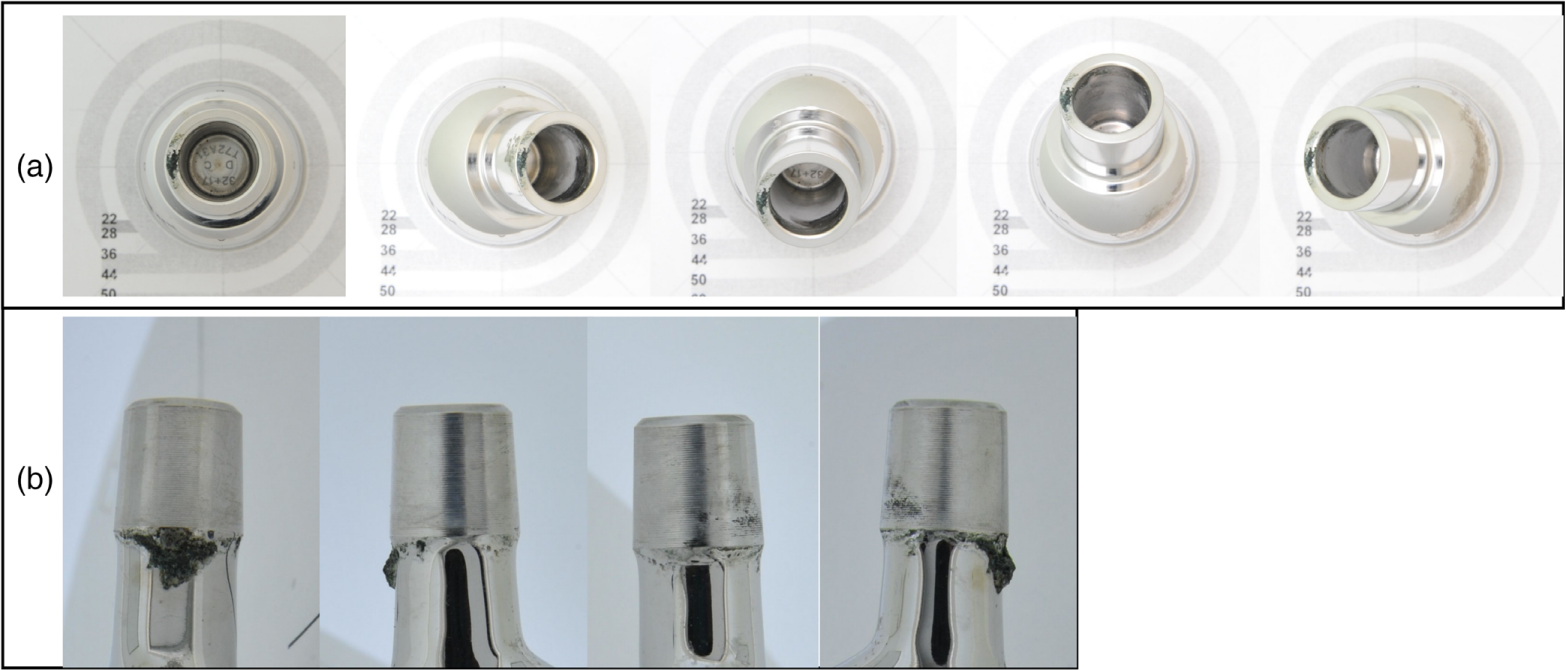
Visual scoring method. Example images of the five standardized positions of the femoral head taper (a) and four standardized positions of the femoral stem trunnion (b) used for visual scoring^11^

Each of the retrieved femoral heads was photographed in five standardized positions that collectively captured the entire femoral head taper surface (Figure 1a). Exposure for each image was optimized for the surface being photographed. For example, tapers, where the engaged surface was more recessed, required increased illumination. Each of the retrieved femoral stems was also photographed in four standardized positions that collectively captured the entire femoral stem trunnion surface (Figure 1b). The high‐quality images from the standard positions for each device component were digitally stitched together allowing the entire surface to be viewed as a whole, flat, single image. The digital images of each component were scored by three independent observers. The observers were able to distinguish damage features while excluding explant removal damage. The observers were blinded to the clinical history, revision diagnosis, and duration of implantation.

Femoral head tapers and stem trunnions were scored independently so that observers did not know which components had been implanted together. The extent of corrosion on the engaged portion of the taper and trunnion surface was assessed by visually inspecting the digital images and scored using two different scoring methods, the 5‐point Anderson Criteria for Corrosion^19^ and the 4‐point Goldberg Criteria for Corrosion and Fretting,^10^ as presented in Di Prima et al.^11^ If there was a discrepancy in scores between different observers for a particular component, the median score of the three observers was used for subsequent analyses.

#### 2.2.2 Digital mosaic method

The entire contact surface of each femoral stem trunnion and head taper component was imaged using custom fixtures to maintain relative positioning between images. Using the Digital Mosaic Method (DMM),^11^ individual image sections were digitally stitched into their respective detailed mosaic of the entire contact surface for all components. Three independent observers identified and manually drew contour boundaries around regions of wear (defined as where the machining marks were damaged or no longer visible) and corrosion (visible grain boundary corrosion or build‐up over the machining marks) using the ImageJ software (NIH, Bethesda, MD). The areas of the identified damage were then added (thereby combining wear and corrosion) to determine the total damaged area, and normalized by dividing by the total contact area for a possible score of 0–100. All images were randomized to ensure that there was no observer bias caused by the assessment of the matching surface.

### 2.3 Profilometry

Surface roughness (*R*_*a*_) evaluations were made for all surfaces using a noncontact optical profilometer (ContourGT‐1, Bruker Corp., Billerica, MA). All measurements were taken using white light and 10× objective and 0.55× magnifier resulting in a measurement area of 1.16 mm by 0.87 mm. This allowed measurements of approximately 46% of the trunnion area.

The components were mounted on the same fixture used for the DMM. Two hundred discrete surface roughness measurements were made for each component. Twenty measurements were made around the circumference at each of 10 longitudinal steps, using an 18° rotation and 1.3 mm step size to capture the average surface roughness for the entire surface. Additionally, surface roughness measurements were made for each taper and trunnion on an unworn section of the surface. To enable roughness measurements of the internal tapers, the taper was filled with liquid silicone (Microset 101FF, Microset Products Ltd., UK) with an 8–32 nut placed at the top of the taper to serve as an attachment point for the casting. After the silicone cured, it was removed from the taper, and measurements were taken from the casted surfaces.

### 2.4 X‐ray photoelectron spectroscopy

The constituents of debris samples from 2 M/M explants were evaluated using XPS (Anderson Materials Evaluation, Inc., Columbia, MD). Debris samples were examined with optical microscopy and metallographic microscopy techniques to identify areas for analysis and to document the appearance of the inhomogeneous and differing deposits. The first debris sample was analyzed at the surface and then at the subsurface via argon etching (100 nm). The sample was then ground into fine particles under isopropyl alcohol and then dried on a sample holder. These particle surfaces were etched to a depth of 100 nm and analyzed in the more homogeneous form. The second sample surface was analyzed in two areas followed by a subsurface analysis in a third area via argon ion etching to depths of 150 and 300 nm. The XPS analysis of the debris was performed over areas of about 0.5 mm^2^ using monochromatic Al K_α1,2_ x‐rays at an energy of 1,487 eV and incident on the sample at an angle of 35° with respect to the plane of the sample. The sampling depth is such that about 70% of the signal comes from the outer 10 nm of the surface materials. Quantitative elemental compositions of the deposit were recorded for each analyzed sample.

### 2.5 Statistical analysis

A multivariate correlation followed by pairwise comparison (*α* = .05) was performed to determine if clinical data had any relationship to corrosion wear and assessment scores.

## 3 RESULTS

This study used both qualitative and quantitative assessment techniques to compare modular connections for M/M and C/M head tapers and stem trunnions. On average, the visual, DMM, and surface roughness assessment methods all indicated that the C/M explants had less evidence of wear and corrosion than the M/M explants (Table 2). There was no difference between the taper and trunnion damage assessment as measured by the Anderson and Goldberg scoring systems for either the M/M or C/M groups. For the M/M group, there was a slightly higher average DMM score for trunnions compared to the tapers. For the C/M group, the tapers had a slightly higher average DMM score compared to the trunnions. The average overall surface roughness *R*_*a*_ and the average *R*_*a*_ for the unworn surface was greater on the trunnion than the taper for both M/M and C/M explants (Figure 3). No correlation was found between any demographic variable and corrosion wear and assessment scores.

**TABLE 2:** Average wear and corrosion assessment scores

	Metal/metal modular connection		Ceramic/metal modular connection	
	Femoral head taper	Femoral stem trunnion	Femoral head taper	Femoral stem trunnion
DMM (%)	42 ± 33 (11–99)	50 ± 32 (11–100)	31 ± 10 (19–51)	10 ± 8 (1–24)
Anderson corrosion score	3 ± 1.55 (1–5)	3 ± 1.62 (1–5)	2 ± 0.46 (2–3)	2 ± 0.46 (2–3)
Goldberg corrosion score	3 ± 1.2 (1–4)	3 ± 1.35 (1–4)	2 ± 0.46 (2–3)	2 ± 0.46 (2–3)
Average surface roughness (*R*_*a*_, μm)	0.87 ± 0.36 (0.33–1.62)	2.67 ± 2.38 (0.56–8.64)	0.71 ± 0.23 (0.53–1.17)	2.30 ± 1.44 (0.43–4.39)
Average surface roughness for unworn surface (*R*_*a*_, μm)	0.65 ± 0.29 (0.16–1.07)	1.89 ± 1.07 (0.25–3.36)	0.60 ± 0.56 (0.20–1.87)	2.12 ± 1.33 (0.17–3.41)

*Note*: Scores are presented as the mean rounded to the nearest whole number from the three independent observers averaged for each implant type ± *SD* and (range) from all three independent observers. Surface roughness measurements are presented as average ± *SD* and (range) for each femoral component within each material group.

**FIGURE 2:**
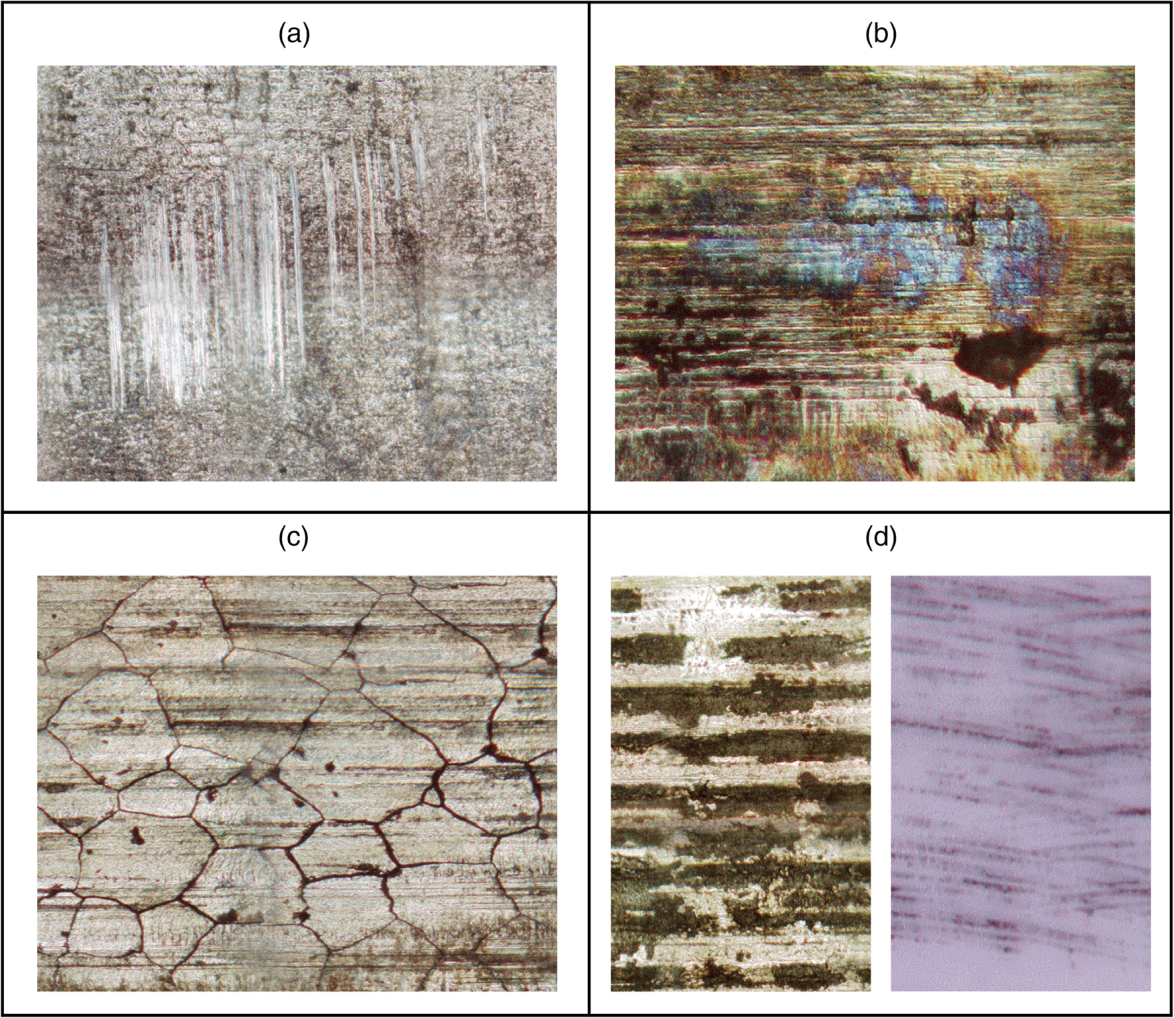
Types of damage features. The four main types of damage features observed on the femoral head tapers and stem trunnions were fretting (a), chemical dissolution of surface oxides (b), intergranular corrosion (c), and surface striations for the M/M and C/M implant surfaces (d)

**FIGURE 3:**
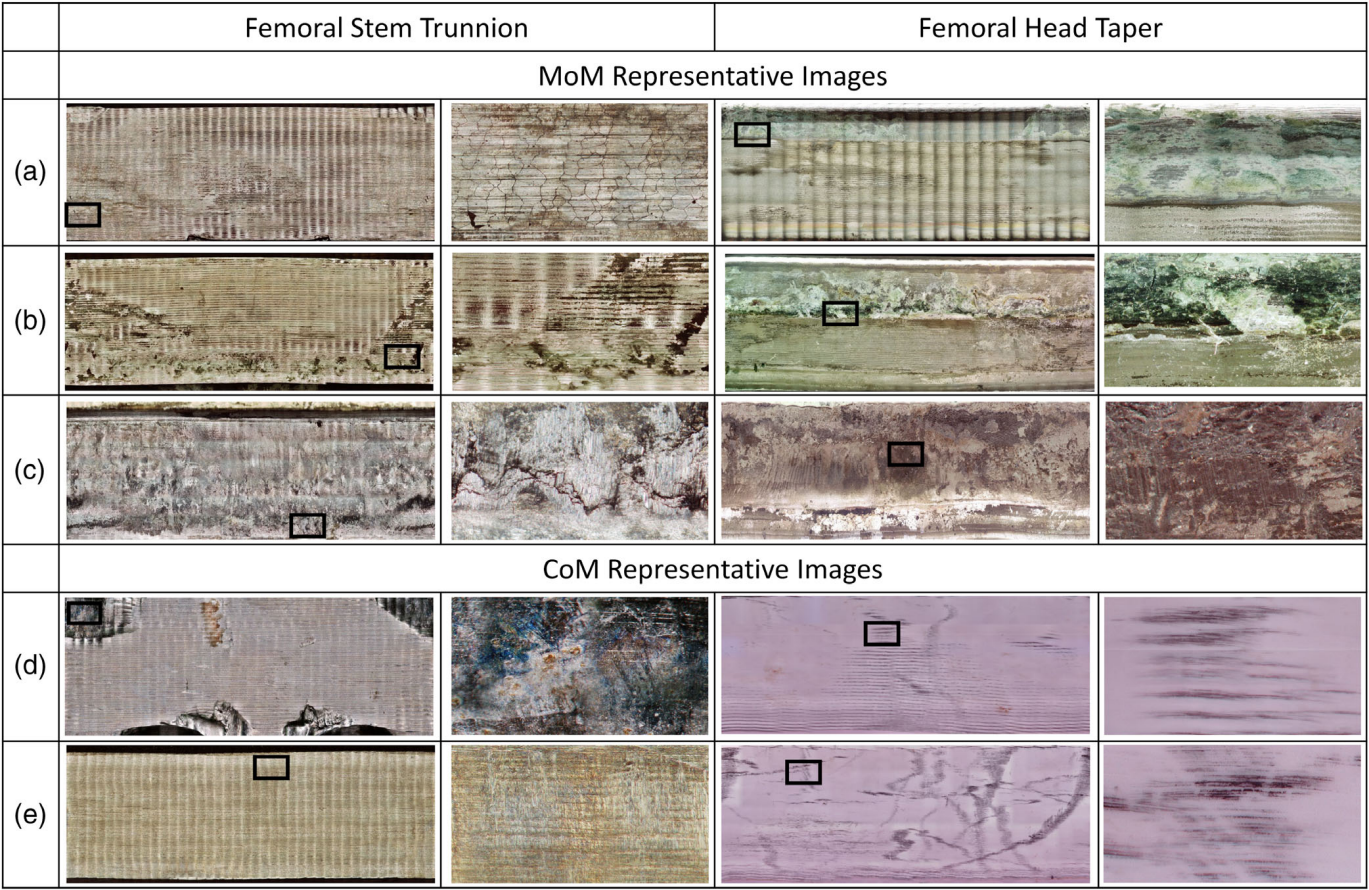
Sample digital mosaic method (DMM) images and damage features. The DMM was used to capture the femoral stem trunnion and head taper for all explants. Each row (a–e) corresponds one set of taper/trunnion DMM images for a given explant system. The width of each full DMM image corresponds to the diameter of the femoral stem trunnion and head taper. The magnified area is indicated by the black box in each full DMM image and captures 10% of the width of each full DMM image

The damage features observed can be categorized into four general groups: fretting, chemical dissolution of surface oxides (dissolution), intergranular corrosion, and surface striations (Figure 2). In general, there was more evidence of wear mechanisms and corrosion on the trunnions than on the tapers. Although the M/M explants exhibited more evidence of wear mechanisms and corrosion, the C/M explants also showed evidence of wear, corrosion, and metal transfer. The wear mechanisms and corrosion were visually different between the two types of explants (Figure 3). All 10 M/M trunnions had some evidence of wear and/or corrosion. Evidence of wear was observed on 5 of the 10 M/M explants. Fretting (7 of 10), surface striations (4 of 10), dissolution (5 of 10), and intergranular corrosion (2 of 10) were also observed on trunnions. Seven of 10 M/M tapers had evidence of wear and/or corrosion. Wear (3 of 10), fretting (3 of 10), surface striations (4 of 10), and dissolution (3 of 10) were also observed on tapers. All 8 C/M trunnions also had evidence of wear and/or corrosion. Wear (1 of 8), fretting (5 of 8), surface striations (4 of 8), dissolution (3 of 8), and intergranular corrosion (1 of 8) were observed on trunnions. Only 4 C/M tapers had evidence of wear, which looked different from the wear on the M/M samples. For explanted components that had evidence of wear and/or corrosion, two or more types of damage features (wear, fretting, surface striations, dissolution, and intergranular corrosion) were commonly observed. Figure 3 and Table 3 contain representative M/M and C/M images and the corresponding DMM, Goldberg Corrosion Scores, and Anderson Corrosion Scores.

**TABLE 3:** Wear and corrosion assessment for samples from Figure 3

		Femoral stem Trunnion			Femoral head taper		
		DMM (%)	GCS	ACS	DMM (%)	GCS	ACS
Metal/metal modular connection	A	85 ± 20.7	4	4	68 ± 13	4	5
	B	56 ± 28	2	2	88 ± 9	3	3
	C	89 ± 15	4	5	99 ± 0.7	4	4
Ceramic/metal modular connection	D	14 ± 3	2	2	25 ± 5	2	2
	E	2 ± 1	2	2	37 ± 5	2	2

*Note*: DMM is reported as mean ± *SD* while Goldberg Corrosion Scores (GCS) and Anderson Corrosion Scores (ACS) represent the median score of the three independent observers.

**FIGURE 4:**
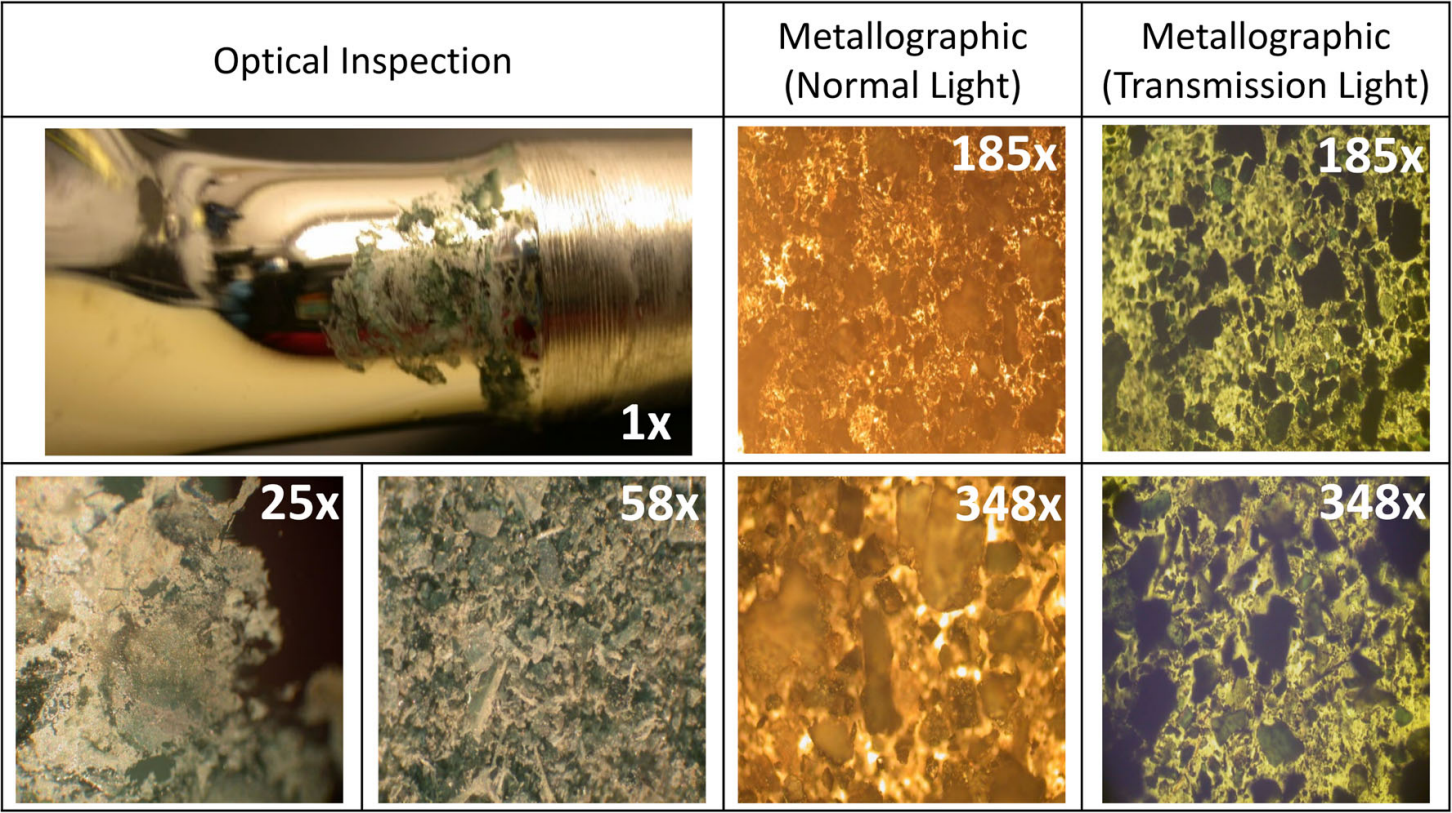
Images of the second explant examined by x‐ray photoelectron spectroscopy (XPS). Wear debris visually inspected using optical and metallographic microscopy techniques prior to XPS analysis. Both normal light and transmission light fields were used in the metallographic microscope images. Width (mm) is noted for each microscopy image

The first M/M debris sample used for XPS analysis included both lighter and darker areas composed of varying concentrations of near white, green, and black materials. The second M/M debris sample had a greener appearance, though off‐white material was also present. Photographic and microscopic images of the debris of the second explant analyzed by XPS are shown in Figure 4, including optical and metallographic microscopy images. These images show the range of size and coloration for the debris particles.

The XPS analysis revealed that the deposits contained a mix of organic and inorganic materials. The primary inorganic material was Cr_2_O_3_ • *x* (CrPO_4_ • 2H_2_O), where *x* values of 0.245, 0.339, and 0.807 were found in various areas of the debris materials from the two explants. The darker green (nearly black) particles had lower *x* values than the lighter green areas. Chromium sulfide, sulfate, and hydroxide were also found. Molybdenum oxides and hydration products were found to be enhanced relative to cobalt materials in terms of their relative concentration in the CoCrMo alloy. There was remarkably little cobalt in the debris samples. Aged bone minerals and calcium hydroxyapatite were also found in the samples. Some graphitic carbon was found in the surfaces of the particles while sulfides and both chromium and molybdenum carbides were found deeper into the debris particles.

## 4 DISCUSSION

The goal of this study was to present both qualitative and quantitative assessments to evaluate wear mechanisms and corrosion at the modular connection for M/M and C/M head tapers and stem trunnions from explanted THAs. Overall, all qualitative and quantitative assessment techniques demonstrated that the M/M explants had more evidence of wear mechanisms and corrosion than the C/M explants. While this may indicate improved in vivo performance of C/M modular taper‐trunnion connections compared to M/M, all 18 devices caused complications resulting in explantation and revision for each patient. Therefore, it is important to understand both clinical and materials factors at the modular taper‐trunnion connection that may affect clinical outcomes. By looking at the combination of clinical data, visual scoring assessments, optical imaging, profilometry, and XPS for both M/M and C/M explant groups, the performance of M/M and C/M modular taper‐trunnion connections may be elucidated.

While this study was not designed to have matched M/M and C/M data sets, the patient age at implantation, BMI, and time in vivo were comparable (Table 1). Both the M/M and the C/M devices appeared to be properly implanted, as evidenced by the cup abduction and anteversion angles. There were no noticeable differences in the overall clinical outcomes. In addition, there were no apparent correlations with any of the clinical variables (e.g., BMI, gender, etc).

The Anderson method, Goldberg method, and DMM were used to evaluate the condition of the contacting modular surfaces. The Anderson and Goldberg methods were developed to provide the clinician with a simple “real‐time” assessment of the device's condition, thereby allowing rapid clinical decisions during revision surgery. While scores 1–4 in both the Anderson and Goldberg methods use the same criteria, the Anderson method includes an additional criterion that differentiates “5—extreme” from “4—severe.”^10^, ^19^ Despite this additional criterion, the mean scores between the three observers for both methods resulted in the same scores for a given implant and did not differentiate between damage in the tapers and trunnions. This indicates that for these explants, the addition of the fifth criterion in the Anderson method did not provide additional information from what was already captured by the Goldberg scoring method.

The DMM method, however, is designed to provide a more comprehensive, postexplantation, quantitative assessment of the wear mechanisms and damage of the entire surface as part of a root cause failure analysis. Thus, it is natural that DMM will provide different results compared to the Anderson and Goldberg methods, as demonstrated in this study. The DMM method considers both wear and corrosion as equal contributors to the damage score, and provides a quantification of the extent of the damaged surface. The Anderson and Goldberg methods, on the other hand, bias even small amounts of damage, and are binary indicators of damage (e.g., present or not present). While the DMM method avoids bias of small amounts of damage, it is very labor intensive. A more automated approach for determining the extent of wear, coupled with computational models, could be useful to better assess damage and predict explant performance.

For the M/M explants, the DMM method revealed more damage on the trunnion than on the taper. In the C/M explants, the opposite was found (Table 2). This can be explained by the different type of damage for the two explants. In the M/M, the higher surface roughness of the trunnion may be more susceptible to wear against the smoother taper surface.^20^ The peaks of the trunnion's machining marks are constantly in contact with the surface of the taper. As the peaks slide during micromotion, a greater area of the taper surface is contacted, resulting in relatively more damage to the trunnion peaks than the smoother taper surface. For the C/M, the increased damage of the taper could be due to the presence of metal material transfer from the metal stem trunnion to the ceramic head taper; gray striations from CoCrMo transfer are readily visible on the light colored ceramic indicating metal transfer. Similar metal transfer markings have been reported for explanted ceramic THA components.^7^, ^21^, ^22^ While differences in surface roughness values have been reported between the transfer markings and the unaffected surface, the direct cause of such markings is still unknown.

For all explants (M/M and C/M), the trunnion average *R*_*a*_ values were not statistically different (*p* >.05). The same was observed for M/M and C/M taper average *R*_*a*_ values. In both cases, the *R*_*a*_ of the trunnion was significantly greater than that of the taper (*p* <.05). Increased trunnion roughness is consistent with previously published studies.^23^, ^24^ The higher surface roughness of the trunnion may exist by design to compensate for any angular mismatch between the taper and trunnion. The higher roughness of one component, in this case the trunnion, will lead to a greater contact area through the deformation of the machining mark peaks.

The XPS analysis allowed for differentiation between corrosion and wear debris. Wear debris should have a composition, in atomic percent (at.%), close to the bulk material (approximately 57.4 at.% Co, 31.5 at.% Cr, and 3.6 at.% Mo). The XPS analysis found the debris to be composed of materials with much greater concentrations of chromium than cobalt and also relatively greater concentrations of molybdenum than found in the bulk alloy. Chromium can move to the surface of the alloy and is enriched in the surface oxide layer. Chromium and molybdenum tend to also migrate to grain boundaries but form carbides in the bulk of the material. However, near the surface, the more stable form of carbon, graphitic carbon, was observed. This is because carbide formation is not favored at the surface. Argon etching of the debris particles exposed the interior carbides below the surface. The presence of interior carbides suggests that there was some preferential grain boundary corrosion so that grains of the alloy could be pulled out while the interior carbides were unaffected. This grain boundary corrosion is known to result when graphitic carbon, rather than carbides, is prevalent. The very high chromium concentrations and the relatively elevated molybdenum concentrations imply that the debris is primarily the result of corrosion, not wear, acting on the chromium and to a lesser degree on the molybdenum.

The color of the corrosion products is explained by the XPS results. A change in the x value in Cr_2_O_3_ • *x* (CrPO_4_ • 2H_2_O) explains the range of colors observed. Chromium oxide (Cr_2_O_3_) is green and the orthophosphate is light violet with some dependence on the degree of hydration. Darker green or black particles had greater concentrations of molybdenum and cobalt. The presence of chromium hydroxide and molybdenum oxyhydroxide indicate an alkaline environment. Sulfides were found beneath the surface of the debris particles, while the surface had sulfides and chromium sulfate. These sulfur‐containing materials are corrosive. The debris sample from the first explant had more sodium and less sulfur while the debris sample from the second explant had no detected sodium and more sulfur. This is one of several indicators that the environment in the body can vary significantly and affect the corrosion products.

The primary limitation of this study is that it is based on THAs that were all explanted. All of the devices had to be removed for various reasons, and therefore are not necessarily representative of functional devices. Additionally, this explant study is forensic, and does not reveal all aspects of the device history. Little is known regarding how the devices were implanted with respect to their orientation and seating in the bone, and nothing is known regarding the methods used to explant the devices. This makes it difficult to differentiate between in vivo damage due to wear and corrosion and damage due to explantation. Further, we have no knowledge of patient behavior, such as activity level, or patient biology. The explanted devices represent a range of different designs and manufacturers, which adds variability. Different assessment techniques have been used to evaluate the modular connection of THAs with metal and ceramic head tapers; however, the materials are known to have different damage features, supported by the observations in this study. This makes comparing wear mechanisms and corrosion for head tapers of different materials highly complex. While this study was able to provide an in‐depth comparison between wear mechanisms and corrosion of M/M and C/M THAs the sample size for each device type is fairly small. This small sample size may have resulted in different wear mechanisms and corrosion scores than other published literature. However, it can be difficult to discern what observers in other studies consider to be damage (i.e., volumetric loss vs. Goldberg/Anderson score). The combination of visual scoring methods, DMM, profilometry, and XPS used to assess wear mechanisms and corrosion in this study were aimed to provide a better sense of the wear mechanisms and corrosion as a whole for this subset of explants. A larger sample size could allow for more definitive conclusions about wear and corrosion in the different device types.

The results of this explant study suggest that investigating wear mechanisms and corrosion for modular taper‐trunnion connections is a worthwhile area for future investigation. More comprehensive analyses of corrosion and wear debris/deposit and metal transfer for a larger sample size could reveal more about the origins of the damage. A controlled bench study investigating angular mismatch between the taper and trunnion and the effect of surface roughness could help to understand potential wear mechanisms and corrosion mechanisms and increase understanding of performance of modular tapers in THA.

## Data Availability

The data that support the findings of this study are available from the corresponding author upon reasonable request.
